# Supplementary material for: The Impact of HPV Diagnosis and the Electrosurgical Excision Procedure (LEEP) on Mental Health and Sexual Functioning: A Systematic Review
Source: Cancers (Basel). 2023 Apr 10;15(8):2226. doi: 10.3390/cancers15082226 (PMC10136821; doi:10.3390/cancers15082226)
Supplement: Supplementary file 1 [file cancers-15-02226-s001.zip › cancers-2257041-supplementary.pdf]

Table S1. PICO questions.

	Observational studies	Interventional studies
**Patients**	Women patients with a positive HPV Test; suffering from genital lesions linked to HPV infection; with abnormal cervical cytology; with low-grade cytological abnormalities; undergoing LEEP procedure; diagnosed with HR-HPV; diagnosed with a borderline Pap smear; undergoing a Pap smear, viral typing, or colposcopy; diagnosed with CIN-2 or CIN-3 lesions and planned for LEEP; reporting sexual dysfunction following LEEP procedure	Women patients with positive HPV Test, abnormal Pap smear; under 65 years of age and over 18 years of age
**Intervention**	Patients receiving a positive HPV Test, abnormal Pap smear, referred to or underwent LEEP procedure, viral typing, or colposcopy; comparison of patients' perceptions at different time intervals	Didactic materials/ presentations regarding HPV infection
**Comparison**	Women with negative HPV Test, without abnormal Pap smear, not undergoing LEEP procedure; measurements in time	Self-administered questionnaires (pre, post) the educational intervention, questionnaires focused on well-being after the HPV diagnosis, comparison of notification of an abnormal Pap smear result via HCP and via medical platform, comparison patients' feelings about HPV Test and Pap smear
**Outcome**	Impact on anxiety, cervical cancer worries, depression, HPV-related shame, quality of life, and sexual function (evaluated by clinical scales)	Impact on anxiety, cervical cancer worries, depression, HPV-related shame (clinical scales)

Table S2. The Impact of HPV diagnosis on anxiety, depression and psychosocial quality of life.

Author, year, country	Study design	Characteristics of the research group	Sample size (N), Average age	Intervention	Study methodology Measurements of effects	Conclusions
Alay et al., 2020, Turkey [31]	Cross- sectional Observational Study	The research involved 80 sexually active women who had been diagnosed with a High-Risk HPV infection. These patients were allocated into four groups, which were as follows: group 1 consisted of patients who were HPV 16/18-positive and had normal cytology; group 2 consisted of patients who were HPV 16/18-positive and had abnormal cytology; group 3 consisted of patients who were non-16/18 HPV-positive and had abnormal cytology, and group 4 consisted of patients who were non-16/18 HPV-positive and had normal cytology	N = 80, Average age-38.3	At the initial clinical visit and again 2 months later, the patients' sexual function and anxiety status were evaluated using the Female Sexual Function Index (FSFI) and Beck Anxiety Inventory (BAI), respectively	BAI, FSFI	The diagnosis of HPV 16/18 has a more adverse impact on women's overall Female Sexual Function Index (FSFI) and desire scores than other genotypes of HPV
Andreassen et al., 2019, Norway [45]	Cross- sectional Observational Study	Females between the ages of 34 to 69 residing in one of the four implementation counties were assigned to one of the following groups: (a) High-Risk HPV testing with a five-year screening interval (followed by cytology if High- Risk HPV positive) or to (b) cytology with a three-year screening interval (followed by High- Risk HPV testing in case of low-grade cytology)	N=1008, Average age-51.2	Women received a structured questionnaire including the Patient Health Questionnaire-4 for anxiety and depression by postal mail 4 to 24 months after they had been informed about their last screening test result	PHQ-4	The prevalence of anxiety and depression, as measured by the PHQ-4 scale, was nearly the same in both the cytology and High-Risk HPV arms. Of the participants, 73% had normal scores, 22% and 21% had mild scores, and 5% and 6% had moderate to severe scores. Nonetheless, no discrepancy in long-term (4 to 24 months following the screening) anxiety and depression scores was found between the colposcopy and High-Risk HPV test group, which suggest that the anxiety doesn’t depend on the method of screening

Atallah et al., 2022, Lebanon [25,26]	Prospective Observational Study	Women suffering from genital lesion linked to HPV, or undergoing a Pap smear, viral typing, or colposcopy	N=118, Average age-41.2	The study included participants who were categorized into 7 different diagnostic groups based on HPV-related pathology: (1) normal pap smear, (2) Pap smear showing atypical squamous cells of undetermined significance (ASCUS), (3) positive HPV test result, (4) CIN-1 lesions, (5) CIN-2/3 lesions, (6) invasive cervical cancer, and (7) genital warts. The participants were given HIP and HADS questionnaires, along with 6 questions aimed at collecting sociodemographic data	The study collected data through the use of the HIP and HADS questionnaires, as well as through 8 additional items aimed at gathering sociodemographic information such as age, area of residence, religious affiliation, civil status, education level, professional status, monthly income, and field of work	The results show that women tested positive for the HPV virus are describing concerns and worries. Except for women with genital warts, the study population reported a strong emotional impact from their HPV-associated pathology, while women with genital warts expressed greater concerns regarding the risk of transmission and the potential impact on their partners
Chadwick et al., 2022, Australia [11]	Prospective Observational Study	Female individuals residing in Australia aged 25-74 who had undergone cervical screening as part of the updated National Cervical Screening Program were eligible to participate	N=915, Average age-38.5	Participants had to fill in questionnaires measuring the anxiety, and emotional and general distress.	STAI, GHQ-12, PEAPS, CSQ	After 2 years of the implementation of the renewed National Cervical Screening Program in Australia, individuals who reported testing positive for HPV still reported significantly higher levels of anxiety, concern,

				Secondary outcomes included psychosexual distress, cancer worry and understanding of HPV tests results		and specific distress about their test results compared to those who reported testing negative
Ciavattini et al., 2021, Italy [53]	Prospective Observational Study	Women with a positive HPV test or abnormal cervical cytology result in the previous 12 months	N=1003, Average age- 38	A survey was created online to assess various aspects, including the demographic characteristics of participants, the primary emotions experienced during diagnosis and treatment, significant changes in lifestyle, the effect on social life, and the sufficiency of information provided to patients	Questionnaire not validated; questions are attached to the research paper	The study findings suggest that anxiety, worry, and fear were the most commonly reported emotions among participants. These emotions persisted throughout all phases, especially during the waiting periods, and decreased after the final phase when calmness and optimism were more commonly reported. As a result, receiving a positive HPV test or an abnormal Pap smear result significantly influenced women's lifestyle, habits, and social life. Participants made efforts to enhance their immune system, take extra precautions in sexual practices, make dietary changes, and modify their sexual and intimate relationships
Clarke et al., 1996, USA [54]	Cross- sectional Observational Study	Men and women with positive HPV result	N=454, Average age- not given	The self-administered questionnaire given to participants consisted of almost 80 questions that covered four major areas: medical history, health-care experiences,	Not validated Questionnaire	This survey's results suggest that the emotional impact of HPV infection can be significant, causing upheaval and anxiety for those affected. Initial reactions for positive test included anger, depression, isolation, shame, and guilt. Following their initial diagnosis, 86% of participants noticed that their infection affected their ability to approach a new

				personal impact, and general demographic information. Participants were specifically asked to provide feedback on how HPV affected them both at diagnosis and within the last 12 months, if applicable		partner, while 73% reported being less spontaneous in their sexual activity, and 72% reported having sex less frequently
Conaglen et al., 2001, New Zeland [46]	Prospective Observational Study	Male and female patients attending the Hamilton Sexual Health Clinic, New Zealand	N=141, Average age-22	Participants in the study completed a self-administered questionnaire at baseline and again after 4 weeks. After the study period, the participants were grouped into three categories for analysis: (1) those diagnosed with first episode genital warts based on clinical diagnosis, (2) those with conditions other than genital warts, and (3) those who were asymptomatic and had negative STI screening tests	GHQ, IAS, IIEF, BISF-W	No significant difference was found between the groups, and there was also no significant change in scores during the follow-up period

Dodd et al., 2020, Australia [33]	Prospective Observational Study	Women between the ages of 25 and 74 living in Australia who have received cervical screening since December 1st, 2017	N=1004, Average age-45	Participants completed sociodemographic, clinical information and questionnaire stating anxiety and general distress	STAI, GHQ-12, PEAPS, CSQ	Women who tested positive for HPV reported significantly higher levels of anxiety, general distress, concern, and distress about test results than women who tested negative for HPV
Drolet et al., 2012, Canada [7]	Cross- sectional Observational Study	Women who received a first or repeat abnormal smear result and a control group of women with a normal smear result	N=952, Average age-31.2	The participants were instructed to fill out three sets of self-administered questionnaires: one at the time of recruitment and two more at 4 and 12 weeks later. The questionnaires covered a range of topics, including socio-demographic characteristics, sexual activity, method of communication for the smear result (in person, by letter, or by telephone), comprehension of information provided, and stressful life events in the past 12 months. The questionnaires also included questions about the utilization of	STAI, HIP, SF-12, VAS, EQ-5D	The receipt of an abnormal result was found to have a significant impact on anxiety levels, although anxiety decreased over time for most women. Nonetheless, 35% of women had clinically significant anxiety at the 12-week mark. This group of women tended to have a lower socio-economic status, reported a lack of full comprehension regarding their test results, and perceived themselves to be at higher risk for developing cancer

				healthcare resources for follow-up of the abnormal smear and the perceived risk of developing cervical cancer in the next 10 years		
Fei Ngu et al., 2018, China [22]	Randomized Controlled Study	Chinese women who had enrolled in another study entitled "A randomized controlled trial comparing concomitant HPV-cytology testing with conventional cytology testing for the detection of high-grade CIN in primary cervical cancer screening in Hong Kong (COCY study)", and who had normal cytology and a positive HPV test result	N=121, Average age-47.52	The study randomly assigned participants to either a leaflet group, which received a written HPV factsheet, or a counseling group, which received a didactic HPV presentation in person along with the factsheet. The participants' psychological states were evaluated through self-administered questionnaires at three time points: before the intervention, within a week after the intervention, and 6 months after the intervention. The main measures of the study were psychosocial well-being, including	HADS, CSQ	At all time points, there was no significant difference in psychosocial well-being between the two groups. Over time, cervical cancer worry and anxiety decreased regardless of the educational interventions. HADS-A scores showed a decreasing trend after the educational interventions, and this trend persisted for 6 months in both the leaflet and counseling groups. However, depression among women (as measured by HADS-D) significantly improved only in the counseling group at 6 months after education compared to before education

cervical cancer worry, anxiety, and depression, screening-related anxieties, HPV-related shame, as well as knowledge of cervical screening and HPV. There was no significant difference in the psychosocial well-being between the two groups at all time points. Irrespective of the two educational interventions, cervical cancer worry and anxiety decreased over time. The Hospital Anxiety and Depression Scale-Anxiety (HADS-A) scores showed a decreasing trend with time after the educational interventions, which was maintained at 6 months in both the leaflet and counseling groups. However, women's depression, as

				measured by HADS-Depression (HADS-D), was significantly improved at 6 months compared to before education in the counseling group only		
Ferenidou et al., 2012, Greece [50]	Cross- sectional Observational Study	Women who had a previous diagnosis of HPV, especially with the genotypes associated with cervical cancer; proceeded to the gynecological outpatient clinic of “Aretaieion” Hospital in Athens, Greece	N=51, Average age-36	Participants completed a questionnaire that consisted of three parts. The first part gathered demographic and clinical data, as well as information on sexual and mental health. The participants were also asked to provide their level of knowledge regarding HPV. The second part of the questionnaire aimed to explore the impact of HPV diagnosis on women's mental health by asking them to describe their feelings following diagnosis. The third part consisted of the	SCSF, A survey was conducted which consisted of 12 distinct items to assess the emotional responses and reactions of participants after their HPV diagnosis	Following an HPV diagnosis, individuals may experience a negative impact on their self-esteem and significant relationships, as well as fears and worries related to their physical health. Furthermore, several sexual dysfunctions may arise, including reduced sexual desire and pain during sexual intercourse

				Sexual Concerns Scale for Women		
Fielding et al., 2017, United Kingdom [27]	Retrospective Observational Study	Women who had low-grade cytological abnormalities identified in the National Health Service Cervical Screening Programme were randomly assigned to either cytological surveillance or initial colposcopy	N=3199, Average age-not given	Women were requested to complete psychosocial questionnaires via mail at 12, 18, 24, and 30 months after their recruitment	HADS, POSM	On average, women with low-grade cytology who are managed by colposcopy have a lower level of psychosocial morbidity during follow-up, compared to those managed by cytological surveillance. Although the difference is modest, any new screening strategies that include a surveillance pathway, such as primary HPV screening, need to ensure that adequate information and support are provided to women
Filiberti et al., 1993, Italy [51]	Prospective Observational Study	Women with a positive HPV test or abnormal cervical cytology result	N=51, Average age-not given	The questionnaires were designed to investigate whether the participants' sexuality was affected negatively, remained unchanged, or improved after treatment. The participants were also asked to provide reasons that might explain any changes in their sexuality; in-depth interview with psychologist	Qualitative face-to-face interviews; IPAT, CDDQ, ASQ	The data collected on sexuality of patients with the disease suggested that it can negatively impact their sexual desire and lead to unpleasant experiences, causing emotional suffering. Many of these patients attribute their disease to sexual activity. Although pain during sexual intercourse was not frequently reported by patients, it was not considered a cause of their sexual impairments. Anxiety and depression tests did not indicate any specific disorders in these patients. The percentage of psychopathological scores in these patients was found to be between those of "healthy women" and "cancer patients"
Garces-Palacio et al., 2019, Colombia	Prospective Observational Study	The study included women with a first time ASCUS cytology reported within three months of recruitment	N=394, Average age-39.9	Participants completed survey with psychosocial	HIP	There were no differences between the groups in terms of improvement in knowledge, self-

[43]				and knowledge measures at enrollment (the survey was constituted by several validated scales and individual questions), two weeksafter triage test results, and 1 year thereafter to assess cervical cancer and HPV knowledge, self-esteem, anxiety and HPV impact		esteem, anxiety, and HPV Impact Profile (HIP). At the beginning of the study, 31.4% and 32.7% of participants had state anxiety and trait anxiety, respectively. These percentages decreased to 10.7% and 13.3% in the final survey. In the second survey, HPV-positive women had worse HIP scores than HPV-negative women
Heinonen et al., 2013, Finland [8]	Prospective/ Retrospective Observational Study	Women referred for outpatient diagnostic colposcopy at the Department of Obstetrics and Gynecology of the Helsinki University Hospital between September 2007 and June 2010 and women who had been treated for cervical dysplasia in the year 2000 were identified from hospital records	N=446, Average age-43	Participants were mailed the HRQoL and STAI Questionnaires to evaluate the anxiety of women	HRQoL, STAI	Anxiety and slight impairment in psychosocial components of HRQoL were associated with abnormal cytology and referral for colposcopy. However, overall HRQoL was not reduced. Impaired HRQoL was associated with high anxiety levels at baseline. Previous treatment for cervical dysplasia was not found to be associated with impaired overall HRQoL
Hsu et al., 2018, Taiwan [34]	Prospective Observational Study	Women visiting a gynecologic clinic in southern Taiwan for their initial follow-up appointment after being diagnosed with HPV were enlisted for the study	N=70, Average age-not given	Two questionnaires were administered to women to assess the level of distress they experienced during the follow-up examination procedure after receiving their	PEAPS, PAIS-SR	During their first visit, 20% of the women reported experiencing emotional distress. Analyses using mixed-effects models revealed that a trajectory of psychosocial adjustment occurred from one to six months after HPV diagnosis, which encompassed health care orientation, sexual relationships, and psychosocial distress.

				abnormal PAP smear results and positive HPV status		However, a significant decline in health care orientation was observed from six to twelve months
Jentschke et al., 2020, Germany [57]	Cross- sectional Observative Study	Women with at least one abnormal Pap smear	N=3753, Average age-31.8	The survey was carried out in two phases in May and June 2018 in Germany, and participants were exclusively approached through an online campaign. The campaign employed various channels, including display marketing (banner), Search Engine Marketing (e.g., Google search), and link-sharing through the prevention and education campaign. Participants had the option to complete the questionnaires online	37-item survey including the "Impact of Event Scale-Revised"; Cervical Dysplasia Distress questionnaires	Regardless of the severity and understanding of the results, abnormal findings in cervical cancer screening can affect a patient's psychological well-being
Johnson et al., 2011, United Kingdom [28]	Randomized Controlled Study	The study population consisted of women who participated in the Trial of Management of Borderline and Other Low-grade Abnormal smears, a randomized controlled trial to determine the most effective and efficient management for women with	N=2842, Average age-30.8	Participants received mailed questionnaire. Women were asked to respond to each question in relation to how	HADS	There was no association between HPV status and anxiety among white women. Among non-white women, anxiety was less common among HPV-positive than HPV-negative women; among non-smokers, cancer worry was more

		low-grade abnormal cervical cytology (borderline nuclear abnormalities or mild dyskaryosis, broadly equivalent to atypical squamous cells of undetermined significance and low grade squamous intraepithelial lesion)		they had been feeling over the previous week		common in HPV-positive than HPV-negative women; the opposite association was observed among ex-smokers
Kitchener et al., 2008, United Kingdom [67]	Prospective Observational Study	Women who underwent routine cervical screening with normal or mildly abnormal cytology	N=705, Average age-28.5	About two weeks after receiving their baseline cytology results, women who were enrolled in the ARTISTIC trial were sent a booklet of questionnaires. Women in the HPV-revealed group were provided with their HPV test results along with their baseline cytology results, whereas those in the concealed group were only informed about their cytology results	GHQ, STAI, SRS	Studies on HPV testing using qualitative research methods have indicated that negative emotions related to the testing process can have an impact on both psychological and psychosexual functioning. However, this impact may not be significant enough to result in GHQ caseness. In women with normal cytology, receiving an HPV test result was linked to a decrease in the SRS
Kwan et al., 2011, China [9]	Prospective Observational Study	Chinese female patients with ASCUS who underwent reflex HPV testing (142 were negative for HPV, while 157 were positive for HPV)	N=299, Average age-36.8	At baseline, self-administered questionnaires involving cancer worry and psychosocial burden topics were given to the participants to	Breast Cancer Worry Scale, STAI-6, HIP	The group of women who tested positive for HPV had noticeably elevated levels of state anxiety, cervical cancer worry, and psychosocial burden in comparison to the group who tested negative for HPV. However, regardless of the HPV test results, all outcome scores showed a

				complete in the clinics both before and after consultation		decrease over time. Nearly 80% of the HPV-positive women attended the recommended colposcopy appointment. After six months, there was no significant difference between the two groups in terms of state anxiety, cervical cancer worry, perceived risk of cervical cancer, and satisfaction with intimate relationships. Additionally, the HPV-positive group continued to experience a higher level of psychosocial burden
Lee et al., 2019, South Korea [44]	Cross- sectional Observational Study	The study examined males with and without genital warts and females with selected HPV-related diseases visiting primary care physicians, obstetricians/gynecologists, urologists, and dermatologists; Females had to experience an HPV-related event within the past 3 months	N=250, Average age- 35	After being diagnosed with an HPV-related disease, participants were given three validated questionnaires that had been translated to Korean and culturally pretested. These questionnaires were administered by the participating physician	HIP, CECA, EQ-5D	The presence of HPV-related diseases in women has a negative impact on their well-being and HRQoL scores. Among women, those with genital warts experienced a more significant psychosocial impact compared to those with other selected HPV-related diseases
Leite et al., 2018, Portugal [21]	Cross- sectional Observational Study	Women diagnosed with high- and low-risk HPV being followed in two major hospitals. The inclusion criteria were: having a diagnosis of HPV, receiving HPV treatment and having a sexual partner	N=194, Average age- 39.8	Participants had to complete a sociodemographic and clinical questionnaire, and were assessed on	HIP, HADS, CECS, ISS, SpREUK	The study found that women with higher levels of psychological morbidity and emotional suppression experienced a greater psychosocial impact on their QOL due to HPV. Furthermore, older

				the psychosocial impact of HPV on quality of life, sexual dissatisfaction, psychological morbidity, and emotional suppression		age, lower education level, and not using condoms were also associated with greater sexual dissatisfaction. The impact of HPV on QOL was associated with sexual dissatisfaction, psychological morbidity, and age. Age, emotional suppression, and condom use were the factors that contributed to sexual dissatisfaction. The study also showed that condom use moderated the relationship between emotional suppression and sexual dissatisfaction
Maggino et al., 2007, Italy [52]	Cross- sectional Observational Study	The experimental group was composed of 36 women who were diagnosed with HPV; during a periodical check-up, the patients were invited to participate in the study; the control group consisted of 36 women	N=72, Average age-33	The study involved distributing three self-evaluation questionnaires (the CBA-20, the SAT-P, and the BISF-W) to a group of 36 women who had been diagnosed with an HPV infection and another group of 36 women who had never been diagnosed with HPV	CBA-20, SAT-P, BISF-W	According to the study's findings, fear and anxiety were the prevalent emotions experienced at the time of diagnosis. Individuals diagnosed with an HPV infection reported higher levels of trait anxiety, obsessions, compulsions, and concerns related to hygiene and unlikely infections
Marlow et al., 2022, United Kingdom [6]	Prospective Observational Study	The study focused on women who had undergone cervical screening between 2016 and 2017 at one of five sites where HPV primary screening had been implemented in 2013	N=1154, Average age-41.2	Follow-up questionnaires were sent to the women six and twelve months after their baseline questionnaire,	STAI-6, GHQ-12; secondary outcomes included not validated questionnaires	The study found that heightened levels of anxiety and distress experienced by women following an HPV-positive result in the context of primary HPV screening tend to subside within six months, and this continues to be the case

				regardless of whether they had participated at the six-month mark		for at least twelve months. However, women who tested positive for HPV continued to be more concerned about developing cervical cancer at the six and twelve-month marks. These findings underscore the importance of early recall to provide reassurance to women who are no longer at higher risk
McBride et al., 2020, United Kingdom [10]	Prospective Observational Study	Women were eligible if they had received one of six possible combinations of HPV and/or cytology test results	N=1125, Average age-41.2	Women opted to take part by returning their completed consent form and questionnaire stating anxiety and general distress	STAI, GHQ-12	The study showed that testing positive for HPV, regardless of whether the cytology result was normal or abnormal, was associated with negative psychological effects in the short-term during routine HPV primary screening. A cross-sectional comparison of women who received their first versus second HPV positive result with normal cytology indicated that anxiety was likely to be short-lived and did not persist for women who were put on 12-month early recall
McBride et al., 2021, United Kingdom [36]	Comparative Qualitative Study	A group of women between the ages of 24 and 63 who had tested positive for HPV with normal cytology were recruited to participate in a qualitative study conducted in England	N=30, Average age-37.5	Participants had to complete the survey, then they could opt-in to be considered for an interview	STAI, CSQ; BIPQ, IPQ-R	Women who received an HPV-positive result with normal cytology experienced various emotional, cognitive, behavioral, and physiological responses. Some of these responses were specific to, or more pronounced in women with high levels of anxiety
McBride et al., 2022, United Kingdom [37]	Cross- sectional Observational Study	Women testing positive for HPV with no abnormal cells (normal cytology)	N=646, Average age-38.26	Potential participants were identified by National Health Service (NHS) staff	STAI, BIPQ, IPQ-R	This study identified latent illness representation profiles that suggest negative beliefs contribute to anxiety in women who test HPV-positive with normal cytology. The

				at two clinical sites. Women were mailed with a survey		characteristics of subgroups of highly anxious women could be used by policymakers to tailor information in routine patient communications
McCaffery et al., 2004, United Kingdom [38]	Cross- sectional Observational Study	Women attending a National Health Service well-woman clinic in central London for routine conventional cervical screening were recruited into the study	N=271, Average age- 32	Participants had to complete a sociodemographic and clinical questionnaire, and were assessed on the psychosocial impact of HPV	STAI, CSQ	Women with normal cytology who tested positive for HPV were significantly more anxious and distressed than women who were negative. Women who tested positive for HPV, regardless of their cytology result, reported feeling significantly worse about their sexual relationships. Specifically, approximately one-third of the HPV-positive women reported feeling worse about their past and future sexual relationships, whereas less than 2% of HPV-negative women reported the same
McCaffery et al., 2005, Australia [56]	Cross- sectional Qualitative Study	Women who had been diagnosed with an HPV infection through a conventional Pap smear were enrolled in this study from various healthcare settings, including general practices, family planning clinics, and specialist gynecologists located in Sydney	N=19, Average age- not given	All participants had received their test results (HPV and smear) and were asked to participate in the interview, which was including following topics: Background demographic info, The following topics were explored among women who had been diagnosed with an HPV	Qualitative face-to-face interviews	The way in which the clinician communicated the diagnosis of HPV and the method of delivery had an impact on the psychological response of women to the diagnosis. The clinician may have a significant role in moderating the psychosocial effects of the diagnosis by choosing an appropriate manner and mode for delivering the HPV diagnosis

				infection on a conventional Pap smear: screening history and background knowledge, understanding of the HPV test, experience of recent Pap test results, experience of HPV diagnosis, understanding and perceptions of results, disclosure of results to others, emotional impact of results, and experience of treatment or follow-up		
McCaffery et al., 2006, United Kingdom [62]	Cross- sectional Qualitative Study	Women participating in HPV testing in England between June 2001 and December 2003	N=74, Average age- not given	All participants who received their test results (HPV and smear) were invited to participate in an interview that covered the following topics: background demographic information, screening history and background knowledge, understanding of the HPV test,	Interview covering topics from the previous section	Women who tested positive for HPV experienced negative social and psychological outcomes, primarily due to the virus's sexually transmitted nature and its connection to cervical cancer. The women reported feeling stigmatized, anxious, and stressed, and were concerned about the implications for their sexual relationships. Additionally, they were worried about disclosing their results to others. Although anxiety related to the infection was widespread, the impact of a positive test varied. The psychological burden of the

				experience of recent Pap test results, HPV diagnosis, understanding and perceptions of results, disclosure of results to others, emotional impact of results, experience of treatment or follow-up, and suggestions for improving cervical screening in the future		infection was influenced by factors such as the women's relationship history and status, social and cultural norms regarding sex and relationships, and their understanding of HPV's key features
McCaffery et al., 2010, Australia [35]	Cross- sectional Observational Study	Women who attended routine cervical screening and received a borderline cervical smear	N=314, Average age- not given	The study randomly allocated patients to one of three groups: human papillomavirus (HPV) DNA testing (n=104), a repeat smear test after six months (n=106), or allowing the patient to choose between the two tests with the aid of a decision-making tool (n=104). Psychosocial outcomes were evaluated through	SF-36, STAI, CSQ, PEAPS	Despite initially having a more negative impact on psychosocial well-being for women who were assigned to HPV triage, this intervention had a more positive impact on their overall psychosocial health compared to repeat smear testing during the entire year of follow-up

				postal questionnaires at various time points during a 12-month period		
Mercan et al., 2019, Turkey [29]	Cross- sectional Prospective Study	Female participants agreed to participate in the study and were enrolled. 67 women had abnormal cytology and/or were positive for High-Risk HPV. The study's control group comprised 66 healthy females of reproductive age who had never tested positive for HPV. The patients who were diagnosed with HPV-related cervical abnormalities had not undergone any prior gynecological treatment for their diagnosis. The patients were assessed for six weeks after being diagnosed	N=133, Average age- 35.14	Three validated questionnaires were used to assess the sexual health of the participant	ASEX, BAI, BDI	Women with HPV were found to have significantly higher ASEX total scores and ASEX sub scores than the control group in the domains of sexual desire, arousal, genital response, orgasmic experience and their satisfaction from orgasm. The results of the study indicate a statistically significant difference in the BDI scores between the experimental and control groups, but no significant differences in BAI scores were observed. The study suggests that HPV-positive female patients experience a significant decline in sexual function, and this decline is not related to depression or anxiety
Monsonego et al., 2011, France [58]	Retrospective Observational Study	The women who participated in the questionnaire had received an abnormal Pap smear result within the 12 months before the study. The countries included in the study were France, Spain, and Portugal	N=1475, Average age- not given	A questionnaire covering basic demographic information and Pap test results was created in English by the WACC Foundation. The questionnaire also included questions regarding how participants were informed about	The questionnaire was developed in English by a WACC Foundation	The findings of the study validate the need for improved management of psychological support when communicating abnormal Pap smear results to women, in order to reduce their anxiety levels. The study showed that women primarily experienced worry upon receiving an abnormal result and during the follow-up period. Additionally, they expressed a desire for more information on cervical cancer

				their abnormal Pap test result, their emotional response upon receiving the news, their level of satisfaction with the support provided by their medical and personal networks, the type of treatment received, and how they obtained additional information about their results. Eleven questions in the questionnaire had pre-determined checkbox response options, with an additional free-text option for participants to provide additional information, if necessary		screening, indicating that there is room for improvement in this area
Mortensen et al., 2010, Denmark [63]	Cross- sectional Observational Study	The study included women who had been diagnosed with varying stages of cervical dysplasia, including those who had undergone conization and those who had not	N=12, Average age- not given	Women were invited to participate in either an individual interview or in a focus group	Qualitative face-to-face interviews	The study found that the participants perceived cervical dysplasia to be a highly distressing condition, and they were anxious about the monitoring process and the potential delay before treatment or regression of the lesions. The women also expressed a fear of cancer that was not

						necessarily linked to the stage of their dysplasia, but rather to their level of understanding about the condition
Nagele et al., 2016, Austria [48]	Prospective Observational Study	The study recruited women who were suspected of having premalignant gynecologic lesions related to HPV from a colposcopy clinic that was based in a university hospital	N=209, Average age-33	Patients were asked to complete three study questionnaires	CDDQ, FoP-Q, SAQ	Women who have HPV-related precancerous genital lesions, particularly those in the vulva, are more likely to experience worries about their sexual health. The study found that there were no significant differences in questionnaire responses among the three groups of patients, except for sexual consequences (measured by the Cervical Dysplasia Distress Questionnaire) and recent sexual activity (measured by the Sexual Activity Questionnaire). It suggests that HPV-related precancerous genital lesions, especially those in the vulva, may raise concerns about sexual health among affected women
Nagele et al., 2019, Austria [47]	Observational Cohort Study	The study involved women who had been diagnosed with premalignant lesions of the cervix, vagina, or vulva that were associated with HPV	N=92, Average age-38.5	Patients filled in validated questionnaires, completed after clinical evaluation (baseline), at 6- and 12- months follow-ups	FoP-Q, SAQ, CDDQ	Fear of progression, psychosocial distress, and sexual activity in women with precancerous HPV-associated premalignant genital lesions seem to be independent from type of treatment
Nahidi et al., 2018, Iran [32]	Cross- sectional Observational Study	37 anogenital warts patients and 37 healthy controls who admitted to Imam Reza Hospital	N=74, Average age-36.9	Evaluation of the psychological symptoms and quality of life, all patients were provided and	SCL-90-R, SF-36	The study showed that anogenital warts is associated with lower social functioning, mental and general health. The results also show that anxiety and depression are common psychopathological findings in HPV positive patients

				asked to complete the questionnaires		
O'Connor et al., 2018, Ireland [59]	Cross- sectional Observational Study	Women found in patient databases in 20 general practices across Ireland and three Dublin-based Well Women Centers	N=3470, Average age- 38.5	Women were asked to fill in received questionnaires; the survey consisted of various questions covering different areas, such as socio-economic status, knowledge about HPV, and women's expected psychological and social reactions to testing positive for HPV, which included feelings of shame, anxiety, stigma, and worry	Set of 10 factual questions about HPV (appendix A)	The study revealed that there are socioeconomic disparities in the expected emotional responses of women from the general population who test positive for HPV. Specifically, women with lower levels of education, those who are not currently employed, younger women, and those who are not married or cohabiting may be at a higher risk of experiencing more severe psychological distress upon testing positive for HPV
Patel et al., 2018, United Kingdom [64]	Prospective Observational Study	Between April 2015 and December 2016, women from across the Midlands (specifically Leicester, Northampton, and Birmingham) were recruited from two settings - colposcopy clinics and the community - to ensure a sample of both women who had and had not experienced abnormal smear results	N=46, Average age- 34	The study offered participants the choice between a one-to-one semi-structured interview or a focus group session. A total of 41 one-to-one semi-structured interviews were conducted with participants (with a mean length of 30 minutes and a range of 17-58 minutes), as well	Face to face interview	Studies have indicated that anxiety related to abnormal cytology and/or treatment is more significant than concerns about HPV status alone

				as one focus group session (38 minutes) consisting of six community participants who did not know each other beforehand		
Pereira-Caldeira et al., 2020, Brazil [60]	Cross- sectional Qualitative Study	Women with a confirmed diagnosis of HPV infection with clinical lesions and subclinical lesions	N=20, Average age- not given	The quality of life was assessed using a semi-structured face-to-face interview consisting of five questions related to the concept of quality of life. The questions were as follows: 1) Do you understand why HPV infection occurs? 2) Has there been any impact on your sexual life since being diagnosed with HPV? 3) Have there been any changes in your daily life, such as work or daily activities, since being diagnosed with HPV? 4) How do you feel about your diagnosis of HPV? 5) Are you experiencing any physical	Qualitative face-to-face interviews	HPV infection has a significant impact on women's quality of life, impairing their sexual, emotional, physical, and everyday functioning. As a result, it can cause significant changes to women's quality of life

				discomfort related to HPV?		
Rask et al., 2019, Sweden [19]	Cross- sectional Observational Study	Women aged 23-65 years, diagnosed with ASC-US+HR-HPV, LSIL+HR-HPV or HSIL+HR-HPV, and without a diagnosis of cervical cancer, were consecutively recruited from a women's health clinic in a county in the southeastern part of Sweden	N=231, Average age-35.1	A questionnaire was sent to the women via mail within a week of receiving their abnormal Pap smear result, which they completed themselves	HADS, HRQoL, FACIT-CD	This study suggests that women generally report good HRQoL after receiving notification of an abnormal Pap smear result, but may experience anxiety. Interestingly, there were no discernible differences in HRQoL or levels of anxiety and depression symptoms between women who were aware of the sexually transmitted nature of HPV infection and those who were not
Rask et al., 2019, Sweden [20]	Single-arm Non-randomized Interventional Study	Women with an abnormal Pap smear result from Swedish cervical cancer screening programme	N=423, Average age-28	A self-administered questionnaire was used to assess the outcomes, which was completed by the women within a week of being notified of their abnormal Pap smear result. The questionnaire was sent to 192 women in the intervention group and 231 women in the comparison group	HADS, FACIT-CD	42.5% of women in the intervention group versus 48.3% in the control group reported anxiety. The study found that there was a significant difference in HPV awareness between the intervention and comparison groups, with more women in the intervention group being aware of HPV, its sexually transmitted nature, and its potential to cause cervical cancer. However, the results suggest that receiving a phone call notification of an abnormal Pap smear result from a trained healthcare provider does not improve women's health-related quality of life or decrease their anxiety levels
Rosen et al., 2010, Canada [40]	Randomized Controlled Study	Participants for the PICCS study were recruited from the Canadian Cervical Cancer Screening Trial, which is a randomized controlled trial comparing the effectiveness of Pap tests versus	N=723, Average age-50.71	Participants had to complete the survey (two experimental and two control	STAI, IUS, NFCS, MUIS, PANAS	Women who received either the long HPV pamphlet or the short control pamphlet and had higher scores in the mentioned scales, showed more anxiety compared to

		HPV tests in screening for cervical cancer. Women between the ages of 30 and 69 from Montreal and surrounding municipalities were enrolled in the CCCaST between Mayrand et al.'s 2006 study and August 2007, and were subsequently recruited for the PICCS study		pamphlet versions); participants subsequently completed measures of HPV uncertainty and anxiety		those with lower scores. Women with higher scores tended to seek more information on HPV, but they may also be susceptible to experiencing more anxiety as factual uncertainties regarding HPV cannot be resolved solely through the provision of additional information
Sakin et al., 2019, Turkey [41]	Cross- sectional Prospective Study	Women who received HPV screening in İstanbul Kartal Dr. Lütfi Kırdar Training and Research Hospital between August 1st, 2017, and November 1st, 2017, were included in the study	N=300, Average age- 42.19	During follow-up visits at week 2, week 4, month 3, month 6, and month 12, the ASEX and FSFI scales were administered to participants, and the results were explained to the patients at each visit	ASEX, FSFI	Statistically significant effects on sexual functioning were found for educational attainment, socioeconomic status, age, employment status, and marital status. However, sexual functioning was not affected by HPV test results (positive/negative) or time from diagnosis
Santos et al., 2019, Portugal [23]	Cross- sectional Observational Study	Portuguese women infected with HPV	N=105, Average age- 39	Participants answered a sociodemographic questionnaire as well as several other specialized questionnaires at the diagnosis appointment, six months after, and 12 months after the diagnosis	HPVQ, PEKQ, HADS, CECS, ISS, ECR-S, SpREUK, RDAS	Women with higher sexual dissatisfaction and an insecure attachment presented lower dyadic adjustment, emphasizing the role of these psychological variables on dyadic adjustment, over time. The results showed that dyadic adjustment one year after diagnosis was predicted by sexual dissatisfaction at diagnosis and six months later. Age contributed positively to sexual dissatisfaction but not to dyadic adjustment. Thus, older women report more sexual dissatisfaction six months after diagnosis

Sénécal et al., 2011, Canada [49]	Prospective Observational Study	Patients with genital warts, either at the first or follow-up visit for an initial or recurrent episode	N=270, Average age- 31.5	Patients were asked to complete study questionnaires three times over a 6-month period: at recruitment, and 2 and 6 months later	EQ-5D	Genital warts were associated with detriments in the EQ-5D domains of anxiety/depression, pain/discomfort and usual activities and general well-being of men and women as reflected by poorer quality of life scores compared with population norms
Waller et al., 2007, United Kingdom [65]	Comparative Qualitative Study	Female students from the London University	N=811, Average age- 33.33	A 2x2 design was used in a web-based survey where information about HPV was manipulated. The design consisted of two factors: awareness of HPV being sexually transmitted vs. no awareness, and awareness of the high prevalence of HPV vs. no awareness	A set of 17 items that assessed different dimensions of stigma, shame, and anxiety were used in this study. These items were modified from the work of Cunningham et al. [76] on other sexually transmitted infections and qualitative research in the HPV domain. Each item was rated on a 4-point scale	If women are made aware that HPV is sexually transmitted, it has the potential to increase their feelings of stigma and shame upon testing positive for the virus. However, if they are also informed that HPV is common, this may help to reduce their feelings of shame and anxiety. Therefore, it is important to strike a balance between raising awareness of HPV's sexually transmitted nature and its prevalence
Waller et al., 2007, United Kingdom [61]	Cross- sectional Observational Study	All women in this study were selected having tested HPV positive with normal cytology at the trial baseline. The recommended follow-up in the trial for such women was a repeat HPV test at 12 months. Women were recruited to the interview study following participation in the second	N=30, Average age- not given	In-depth interviews were carried out with 30 women who were HPV-positive with normal cytology at trial baseline, and attended for a	Face to face interview	Although women often experienced serious negative emotional consequences at the time of their first positive result, these did not generally last during the year between tests once questions about HPV had been resolved. Many women experienced a

		HPV test, and the sample included those who were reported both positive and negative at the second test		repeat HPV test 12 months later	greater emotional impact when they tested positive for HPV for the second time, causing them to overcome their embarrassment and seek support. Immediate colposcopy was preferred by the majority of women, as they desired a speedy resolution and feared the risk of cancer progression, rather than continued surveillance for persistent HPV
Waller et al., 2009, United Kingdom [66]	Prospective Observational Study	A population-based survey was conducted in 2006-2007 on 1081 women aged 25-64 years in the United Kingdom to obtain the data. The women were given information about HPV in phases, and they were asked whether it would make them feel more or less worried and ashamed if they received an abnormal Pap smear result	N=1081, Average age-not given	The study questionnaires aimed to answer the following questions: 1) What percentage of women expect to experience worry and shame if they receive an abnormal Pap test result? 2) Does providing basic information about the connection between cervical cancer and HPV increase anticipated worry and shame after receiving an abnormal Pap test result? 3) How does information on i) HPV transmission and ii) HPV prevalence	Interview covering topics from the previous section	The vast majority of women did not experience an increase in anticipated shame when provided with information about HPV in the context of an abnormal Pap test result. However, nearly one-third of women indicated that they would feel more worried

	affect anticipated worry and shame?

State-Trait Anxiety Inventory- STAI; General Health Questionnaire- GHQ-12; Psychosocial Effects of Abnormal Pap Smears Questionnaire short-form- PEAPS-Q; HPV Impact Profile- HIP; Cervical Screening Questionnaire- CSQ; Brief Illness Perception Questionnaire- BIPQ; Illness Perception Questionnaire- IPQ-R; Intolerance of Uncertainty Scale- IUS; Need for Closure Scale- NFCS; Mishel Uncertainty in Illness Scale- MUIS; Positive and Negative Affect Schedule- PANAS; Hospital Anxiety and Depression Scale- HADS; Symptom Checklist of Sexual Function- SCSF; Cognitive Behavioural Assessment- CBA-20; Satisfaction Profile- SAT-P; Brief Index of Sexual Functioning For Woman- BISF- W; Health-Related Quality of Life- HRQoL; Functional Assessment of Chronic Illness Therapy– Cervical Dysplasia- FACIT-CD; Beck Anxiety Inventory- BAI; Female Sexual Function Index- FSFI; Specific questionnaire for Condylomata Acuminata- CECA; EuroQol-5 Dimension- EQ-5D; Fear of Progression Questionnaire- FoP-Q; Sexual Activity Questionnaire- SAQ; Cervical Dysplasia Distress Questionnaire- CDDQ; Courtauld Emotional Control Scale- CECS; Index of Sexual Satisfaction- ISS; Spiritual and Religious Attitudes in Dealing with Illness- SpREUK; Patient Health Questionnaire-4- PHQ-4; Short Form- SF; Visual Analog Scale- VAS; Psychosocial Adjustment to Illness Scale-SR- PAIS-SR; Process Outcome Specific Measure- POSM; Ask Suicide-Screening Questions- ASQ; Intensive Care Psychological Assessment Tool- IPAT; The 12-item Short Form Survey- SF-12; Sexual Rating Scale- SRS; HPV Knowledge Questionnaire- HPVQ; Papanicolaou Exam Knowledge Questionnaire-PEKQ; Experiences in Close Relationship Scale- Short Form- ECR-S; Revised Dyadic Adjustment Scale- RDAS; Short-Form-36- SF-36; Symptom Checklist-90-R- SCL-90-R; Arizona Sexual Experiences- ASEX; Female Sexual Functioning index- FSFI; Beck Depression Inventory- BDI; Illness Attitude Scales- IAS; International Index of Erectile Function- IIEF.

Table S3. The impact of HPV diagnosis and LEEP procedure on depression/anxiety, quality of life and sexual function.

Influencing factor	Studies evaluating the impact of HPV diagnosis and LEEP procedure on given variable % (n/N) – percent, (n- number of studies where the impact was significant / N- number of all studies evaluating given variable)								
	Anxiety/ depression Quantitative studies	Anxiety/ depression Qualitative studies	Anxiety/ depression All studies	Quality of life Quantitative studies	Quality of life Qualitative studies	Quality of life All studies	Sexual functioning Quantitative studies	Sexual functioning Qualitative studies	Sexual functioning All studies
**HPV diagnosis**	90% (29/32)	92% (11/12)	93% (41/44) [2,6–11,19–23,25–29,31–34,36–38,40,43–47,49–52,54,57,58,60–65,67]	62% (5/8)	100% (1/1)	67% (6/9) [7,8,19,21,32,35,44,49, 60]	90% (9/10)	80% (4/5)	87% (13/15) [11,21,23,29,31,38,41,46–48,50,51,54,60,67]
**LEEP procedure**	0% (0/3)	100% (1/1)	40% (1/4) [15,24,55,62]	-	-	-	28.5% (2/7)	66.7% (2/3)	40% (4/10) [13–15,17,24,30,39,41,42,55]

Table S4. The Impact of the Loop Electrosurgical Excision Procedure (LEEP) on anxiety, depression, quality of life and sexual function.

Author, year, country	Study design	Characteristics of the research group	Sample size (N), Average age	Intervention	Study methodology Measurements of effects	Conclusions
Gaurav et al., 2022, India [14]	Prospective Observational Study	Female patients diagnosed with CIN-2 and CIN3- lesions and planned for LEEP, between October 2020 to March 2021	N=61, Average age- 40.88	Women were interviewed regarding their sexual function before the LEEP procedure and after 6 months while on follow-up with self- structured pre- and post-procedural sexual function questionnaire	Non-standardized questionnaire	Patients with diagnosed CIN-2 lesions, treatment with LEEP did not show a significant change in frequency of intercourse. However, symptoms of dyspareunia and postcoital bleeding, which indirectly affected sexual health, showed significant improvement post LEEP procedure. These findings indicate that the minor cervical trauma caused by such minimally destructive surgical treatment is unlikely to be a cause of organic sexual dysfunction
Giannella et al., 2013, Italy [55]	Observational Comparative Study	Premenopausal and postmenopausal women undergoing LEEP at Cesare Magati Hospital, Scandiano or Cervical Cancer Screening Center, Reggio Emilia, Italy, from January 2006 to March 2011	N=339, Average age- 48.1	The interview was carried out 4 months after LEEP. During these 4 months, all study participants were evaluated only once, after 30 days, to evaluate the surgical outcomes of LEEP. The questionnaire consists of 8 questions, the first two questions concern the psychological impact of cervix disease; the next two questions concern the impact of LEEP on body image; the following two questions pertain to the effects of LEEP on changes in love relationships, while the last two questions relate to the quality of sexual	Non-standardized questionnaire	Multivariate analysis showed that after LEEP in postmenopausal women, significant psychological changes concerning the impact of cervix disease, their body image, interpersonal relationship with their partner and sexual health quality were found. Such observations confirm that the psychological impact of LEEP seems to be greater in postmenopausal than in premenopausal women. Authors speculate that LEEP could cause failure of psychological domains already weakened by menopause

				health, which is assessed based on the frequency of sexual intercourse and sexual satisfaction		
Giovannetti et al., 2022, United Kingdom [42]	Prospective Observational Study	Women who report sexual dysfunction following LEEP recruited from online patient groups	N=64, Average age-not given	Researchers created a self-report survey that consisted of questions about the locations of sexual responses, barriers to sexual health discussions, and the full FSFI was incorporated as a distinct element of the survey. Participants completed the online surveys, and were subsequently interviewed by the research team	FSFI, Non-standardized questionnaire, Interview	Participants reported experiencing a range of symptoms related to altered or loss of cervical sensation during sex, such as numbness, pain with vaginal penetration, decreased lubrication, as well as issues with partners. They also described experiencing a decrease in sexual desire, arousal, and self-confidence, and reported feeling depressed. Participants expressed dissatisfaction with pre-operative care, particularly with regards to inadequate disclosure of possible risks and screening for sexual dysfunctions. Post-operative care was described as minimal, and many participants reported feeling uncomfortable discussing sexual health concerns with their healthcare providers
Hellsten et al., 2008, Sweden [16]	Prospective Observational Study	Consecutive women referred for colposcopy for the first time subsequent to receiving notification of an abnormal cervical smear and underwent LEEP	N=100, Average age-27	The women completed the State-Trait Anxiety Inventory, a psychosexual questionnaire and had one psychosocial interview prior to colposcopy at all three visits (after an abnormal cervical smear and a 6-month and 2-year follow up)	STAI, Face to face interview	Study's outcome shows that women referred for colposcopy after an abnormal cervical smear report experiencing a long-term effect on their sexual functioning, a lower 'spontaneous interest in sex', and a decrease in 'frequency ofintercourse'. However, no differences in sexual functioning were found between women who underwent LEEP and those who did not. Women with higher levels of state and trait anxiety during the first visit had a higher incidence of dyspareunia and negative feelings about sex compared to those with lower anxiety levels. Women with high trait anxiety continued to experience dyspareunia problems at the 2-year follow-up. Both the LEEP and non-LEEP groups experienced a significant decrease in reported scores for frequency of

						intercourse, spontaneous interest, and sexual arousal at 6 months. The most notable variable was a significant decrease in spontaneous interest in sex that persisted at a highly significant low level at the 2-year follow-up
Inna et al., 2010, Thailand [13]	Cross-sectional Prospective Study	Premenopausal women with cervical dysplasia who had undergone LEEP at least 3 months before the assessment	N=89, Average age-41.7	During post-LEEP follow-up visits, the female participants were interviewed once using a questionnaire to assess their sexual function before and after the procedure. The data collected on the frequency of sexual intercourse, presence of dysmenorrhea, dyspareunia, and postcoital bleeding were analyzed using the McNemar test. Similarly, data on specific aspects of sexual function were rated using a 6-point Likert scale and analyzed using the Wilcoxon signed ranks test	Aspects of patients' sexual life were measured by 6-point Likert scale	When interviewed approximately 29.3 weeks after the procedure, it was found that undergoing LEEP in conjunction with other "non-surgical" methods for managing cervical pre-cancer is linked to slight but statistically significant reductions in overall sexual satisfaction, vaginal elasticity, and orgasmic satisfaction. However, there were no significant changes in other aspects of sexual function. These findings suggest that the adverse impact of the LEEP procedure itself on sexual function is minimal, if at all clinically significant
Le et al., 2006, Canada [24]	Prospective Observational Study	20 LEEP and 21 colposcopy patients	N=41, Average age in the colposcopy group- 28.5, while in the LEEP group-32	Comparison of anxiety and distress in patients undergoing colposcopic examinations and LEEP over a 3-month period	HADS, PEAPS, questionnaire focused on the effects of an abnormal Pap test on current sexual relationship using 14 questions on a Likert scale	Of the patients who underwent colposcopy, 81% can be classified as having anxiety and depression, while 65% of those who underwent LEEP can be classified as such. Moreover, patients who underwent LEEP showed significantly better scores on the PEAPS and sexual relationship scales than those who underwent colposcopy

Michaan et al., 2022, Israel [15]	Prospective Observational Study	Female patients undergoing cervical conization	N=55, Average age- 35.1	Women were asked to fill in three questionnaires (FSDS-r, FSFI, HADS) at recruitment, prior to cervical conization, and 6-12 months later	FSDS-r, FSFI, HADS	There were no significant differences observed in the FSDS-r scores before and after conization, with an equal number of patients (29 patients, 53%) reporting sexual distress both before and after the procedure. The FSDS domains and total score remained unchanged before and after conization (26.8 vs. 26.0, p=0.461). However, the percentage of patients indicating an overall sexual dysfunction increased from 49% before conization to 59% after the procedure. A high percentage of patients also reported signs of anxiety on the HADS questionnaire both before (49%) and after (47%) conization
Plotti et al., 2022, Italy [39]	Prospective Observational Study	Women with the diagnosis of LSIL at PAP-smear and HR-HPV positivity with a persistent CIN- 1 (at least for 6 months), confirmed by cervical biopsy	N=136, Average age- 39.4	All women fill in questionnaires to examine anxiety state and sexual function after enrollment and underwent t-LEEP under deep sedation	STAI, FSFI	A better overall anxiety status and a better sexual function after cervical treatment with a statistically significant result was found. A significant decrease of anxiety status in HPV-cleared patients after cervical treatment has been noticed. In non-cleared patients a slight worsening of anxiety status and sexual function after cervical treatment was found
Sparić et al., 2018, Serbia [30]	Cross-sectional Prospective Study	Women with cold knife conization or LEEP of the transformation zone treatment	N=146, Average age- 35.2	Women were interviewed after a follow-up colposcopy visit. Their demographics, treatment and psychosexual characteristics were recorded	BAI, BDI	A study found that 27.4% of women experienced a decrease in their interest in sexual intercourse after treatment compared to before. Women who reported reduced interest in sexual activity post-treatment also had higher anxiety and depression scores and were more concerned about the progression of their illness. Those who experienced post-treatment complications were more likely to report reduced interest in sexual activity and increased anxiety and depression. Women who had abnormal smear test results during follow-up were also more likely to experience higher levels of anxiety. Interestingly, the type of treatment received and the severity of

						dysplasia did not appear to have any significant impact on the women's interest in sexual intercourse or their anxiety and depression scores
Serati et al., 2010, Italy [17]	Prospective Observational Study	Consecutive sexually active women, who underwent LEEP for the treatment of cervical intraepithelial neoplasia	N=67, Average age-36	All women were asked to complete a copy of FSFI questionnaire, at the time of LEEP and after 6 months	FSFI	LEEP for the treatment of cervical intraepithelial lesions doesn't affect women's sexuality, when compared with sexual function before surgery. All FSFI sexual function domains but desire, did not show significant change after LEEP

Female Sexual Distress Scale-Revised- FSDS-r; Female Sexual Function Inventory- FSFI; Hospital Anxiety and Depression Scale- HADS; Beck Anxiety Inventory- BAI; Beck Depression Inventory- BDI; Loop Electrosurgical Excision Procedure- LEEP; State - Trait Anxiety Inventory- STAI; Cervical Intra-epithelial Neoplasia Grade II- CIN-2; Cervical Intra-epithelial Neoplasia Grade II- CIN-3; Psychosocial Effects of Abnormal Pap Smears Questionnaire- PEAPS.

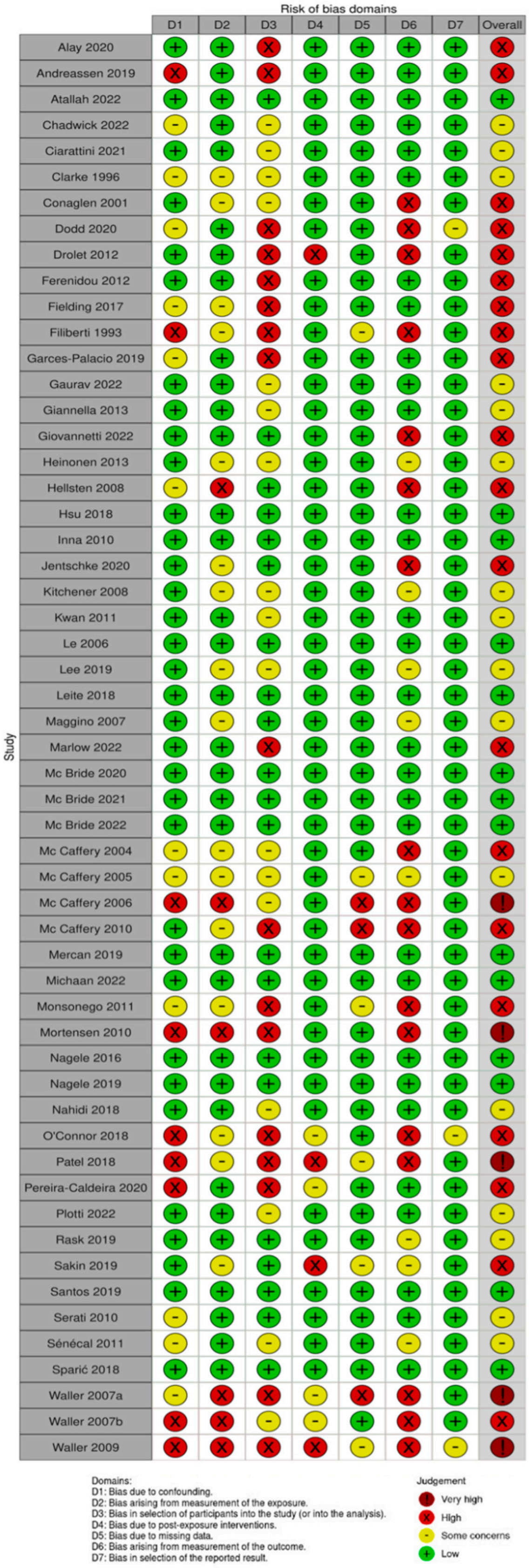
Risk of bias domains
D1
D2
D3
D4
D5
D6
D7
Overall
Study
Alay 2020
Andreassen 2019
Atallah 2022
Chadwick 2022
Ciarattini 2021
Clarke 1996
Conaglen 2001
Dodd 2020
Drolet 2012
Ferenidou 2012
Fielding 2017
Filiberti 1993
Garces-Palacio 2019
Gaurav 2022
Giannella 2013
Giovannetti 2022
Heinonen 2013
Hellsten 2008
Hsu 2018
Inna 2010
Jentschke 2020
Kitchener 2008
Kwan 2011
Le 2006
Lee 2019
Leite 2018
Maggino 2007
Marlow 2022
Mc Bride 2020
Mc Bride 2021
Mc Bride 2022
Mc Caffery 2004
Mc Caffery 2005
Mc Caffery 2006
Mc Caffery 2010
Mercan 2019
Michaan 2022
Monsonego 2011
Mortensen 2010
Nagele 2016
Nagele 2019
Nahidi 2018
O'Connor 2018
Patel 2018
Pereira-Caldeira 2020
Plotti 2022
Rask 2019
Sakin 2019
Santos 2019
Serati 2010
Sénécal 2011
Sparić 2018
Waller 2007a
Waller 2007b
Waller 2009
Domains:
D1: Bias due to confounding.
D2: Bias arising from measurement of the exposure.
D3: Bias in selection of participants into the study (or into the analysis).
D4: Bias due to post-exposure interventions.
D5: Bias due to missing data.
D6: Bias arising from measurement of the outcome.
D7: Bias in selection of the reported result.
Judgement
Very high
High
Some concerns
Low

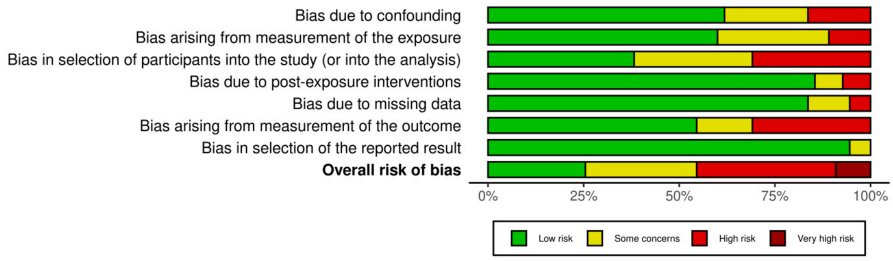

Figure S1. Risk of bias for observational studies separately and summarizing with ROBINS-E tool [6–11,13–17,20,21,24–27,29–39,41–67,73].

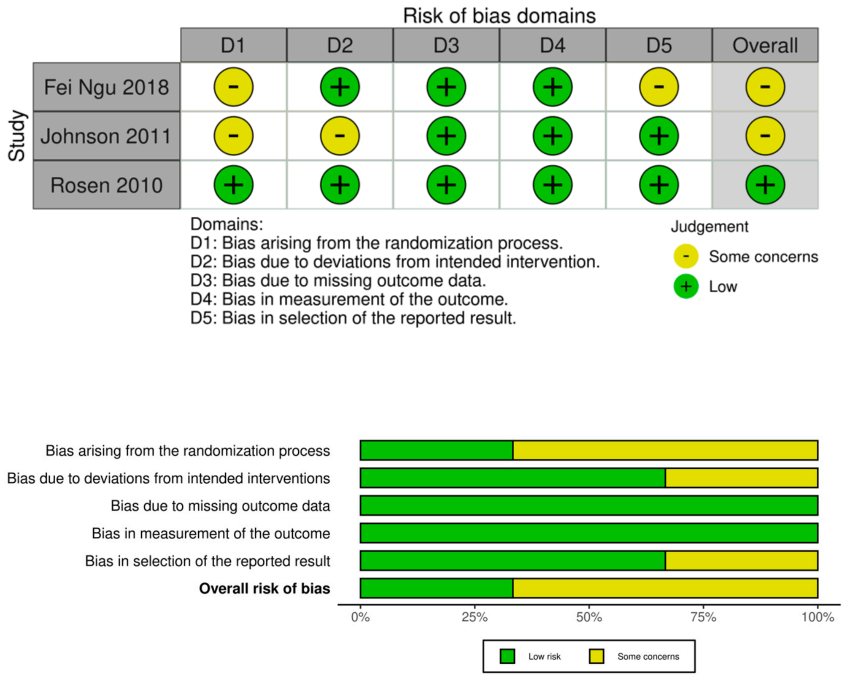

Figure S2. Risk of bias for interventional randomized studies separately and summarizing with Rob2 tool [22,28,40].

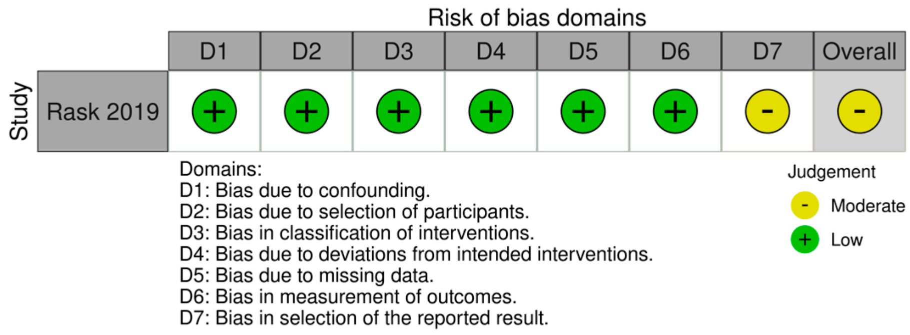

Figure S3. Risk of bias for interventional non-randomized study with ROBINS-I tool [20].
